# From Mangrove to Fork: Metal Presence in the Guayas Estuary (Ecuador) and Commercial Mangrove Crabs

**DOI:** 10.3390/foods10081880

**Published:** 2021-08-14

**Authors:** Andrée De Cock, Niels De Troyer, Marie Anne Forio Eurie, Isabel Garcia Arevalo, Wout Van Echelpoel, Liesbeth Jacxsens, Stijn Luca, Gijs Du Laing, Filip Tack, Luis Dominguez Granda, Peter L. M. Goethals

**Affiliations:** 1 Department of Animal Sciences and Aquatic Ecology, Faculty of Bioscience Engineering, Ghent University, Coupure Links 653, 9000 Ghent, Belgium; Niels.DeTroyer@UGent.be (N.D.T.); Marie.Forio@UGent.be (M.A.F.E.); igarciaa@ifremer.fr (I.G.A.); Wout.VanEchelpoel@UGent.be (W.V.E.); Peter.Goethals@UGent.be (P.L.M.G.); 2 Laboratoire de Biogéochimie des Contaminants Métalliques, Ifremer, Centre Atlantique, CEDEX 3, 44311 Nantes, France; 3 Department of Food Technology, Safety and Health, Faculty of Bioscience Engineering, Ghent University, Coupure Links 653, 9000 Ghent, Belgium; Liesbeth.Jacxsens@UGent.be; 4 Department of Data Analysis and Mathematical Modelling, Faculty of Bioscience Engineering, Ghent University, Coupure Links 653, 9000 Ghent, Belgium; Stijn.Luca@UGent.be; 5 Department of Applied Analytical and Physical Chemistry, Faculty of Bioscience Engineering, Ghent University, Coupure Links 653, 9000 Ghent, Belgium; Gijs.DuLaing@UGent.be (G.D.L.); Filip.Tack@UGent.be (F.T.); 6 Facultad de Ciencias Naturales y Matemáticas, Escuela Superior Politécnica del Litoral ESPOL, Campus Gustavo Galindo, 090112 Guayaquil, Ecuador; ldomingu@espol.edu.ec

**Keywords:** risk assessment, environmental evaluation, consumer health

## Abstract

Mangrove wetlands provide essential ecosystem services such as coastal protection and fisheries. Metal pollution due to industrial and agricultural activities represents an issue of growing concern for the Guayas River Basin and related mangroves in Ecuador. Fisheries and the related human consumption of mangrove crabs are in need of scientific support. In order to protect human health and aid river management, we analyzed several elements in the Guayas Estuary. Zn, Cu, Ni, Cr, As, Pb, Cd, and Hg accumulation were assessed in different compartments of the commercial red mangrove crab *Ucides occidentalis* (hepatopancreas, carapax, and white meat) and the environment (sediment, leaves, and water), sampled at fifteen sites over five stations. Consistent spatial distribution of metals in the Guayas estuary was found. Nickel levels in the sediment warn for ecological caution. The presence of As in the crabs generated potential concerns on the consumers’ health, and a maximum intake of eight crabs per month for adults is advised. The research outcomes are of global importance for at least nine Sustainable Development Goals (SDGs). The results presented can support raising awareness about the ongoing contamination of food and their related ecosystems and the corresponding consequences for environmental and human health worldwide.

## 1. Introduction

Increasing degradation of ecosystems is not only causing biodiversity loss but also affecting the quality of water-related ecosystem services. These services cover a wide range of benefits, including water purification, carbon capture and storage, natural flood protection, and provision of water for agriculture, fisheries, and recreation [1]. For instance, mangrove wetlands have important ecological functions such as coastal protection, salinity buffering, and nutrient transport [2,3,4]. Yet, pressures on these ecosystems are growing worldwide due to anthropogenic activities, with almost 50% of all mangrove wetlands having disappeared over the past 50 years [5,6].

In recent years, pollutants have been found to end up in rivers and eventually in mangrove wetlands as a result of increasing human activities [7]. For instance, many developing countries lack the technology and financial resources to establish municipal wastewater collection and treatment [8], causing the direct discharge of untreated domestic wastewater into the environment. In South America, various anthropogenic activities such as intensive agricultural practices, oil refineries, and metal mining are reported to be important pollution sources of water-related ecosystems [9,10,11,12,13].

Mangrove ecosystems are known as environmental sinks, accumulating various types of contaminants since their sediments act as a chelating matrix for metal(loid)s (Metalloids (e.g., As) are compounds with both metal properties and non-metal properties. As arsenic was investigated in this research as the only metalloid, for reasons of simplicity, it will be included in the term metal throughout the manuscript), pesticides, petroleum, and other contaminants [14,15]. Moreover, biota inhabiting or living in close contact with these sediments are prone to absorb and accumulate these metals [16], thereby introducing them into the food web. The continuous destruction and deforestation of mangrove ecosystems cause the release of previously captured metals from the sediments and potentially accelerates the biomagnification of the elements from one trophic level to the next [15,17]. The uptake of these trace metals can negatively impact the health of aquatic life and also affect human health through the consumption of seafood [18,19,20]. Chemicals, including metals, have been classified as a significant contributor to food contamination [21].

Toxic effects tend to occur when a threshold concentration is exceeded, which is strongly determined by the bioavailability of the considered metal. Therefore, insight into the occurrence, distribution, and bioavailability of different metals is crucial in assessing the toxicity of surface waters worldwide [22]. The metals can potentially induce severe oxidative stress in aquatic organisms [23]. Moreover, several metals have a high affinity with the sulfhydryl group and can inhibit more than two hundred enzymes in the human biological system [24]. Chronic exposure to trace metals causes toxicity in several organs of the human body such as hepatonephrotoxicity and neurotoxicity [25].

Ultimately, the combination of these environmental processes threatens fisheries stocks in the mangrove wetlands [19,26,27]. For instance, mangrove wetlands are harvesting areas for artisanal fishermen who rely almost entirely on subsistence fisheries. Consequently, ecosystem contamination is not only detrimental for aquatic life and human health but also potentially impacts the livelihoods of local fisher communities [28,29].

The assessment of metal distributions in aquatic ecosystems and the identification and management of associated risks and impacts is crucial regarding food safety, the associated socioeconomic aspects of fisheries, and to achieve the United Nations Sustainable Development Goals (SDGs) [9,22,30,31]. Several SDGs are targeted with our and similar studies, including ensuring healthy lives (SDG 3), sustainable water management (SDG 6), sustainable consumption and production (SDG 12), and protecting life below water and on land (SDGs 14 and 15) (Section 4.4). Several studies have investigated the environmental or the human health risk of metals in aquatic ecosystems [32,33,34,35,36]. Those studies reported metal occurrence in several environments and/or organisms and mention the harmful effects on consumer’s health due to metal accumulation in water or biota. However, studies rarely feature an integrated approach of environmental evaluation and consumer risk assessment of metals in the environmental and crab compartments of mangrove ecosystems, in particular in developing countries. Furthermore, those results are often not linked to the SDGs. This study aims to fill this void by analyzing metal accumulation in different compartments of the red mangrove crab (*Ucides occidentalis*) (hepatopancreas, carapax, and white meat) and the environment (sediment, leaves, and water), sampled at 15 stations in the Guayas estuarine ecosystem. The Guayas River Basin in Ecuador has been subjected to growing agricultural and industrial activities during the past few years, and several studies have shown the accumulation of contaminants in the ecosystem [5,10]. *U. occidentalis* is a crab species that resides in the mangroves of the west coast of Latin America, including the estuary of the Guayas river. This red mangrove crab is a traditionally exploited crustacean with high economic importance, being a popular dish in Ecuador [37,38].

There are four specific aims in this research: firstly, to determine the metal concentrations in each compartment (sediment (S), leaves (L), water (W), hepatopancreas (HP), carapax (CP), and white meat (WM)); secondly, to identify the spatial distribution and differences in metal concentrations between the different stations; thirdly, to perform an environmental evaluation based on international and national indices; and fourthly, to perform an intake risk assessment for consumer’s health including the communication of a safety limit for the intake of crabs. Finally, we associated several SDGs with the outcomes of this research. The findings presented in this research can provide useful insights to river managers, policymakers, and other stakeholders at a national and international level.

## 2. Materials and Methods

### 2.1. Study Area

The study area is located in the coastal region in the central–western part of Ecuador at the inner estuary of the Gulf of Guayaquil, which is also known as the Guayas river estuary. The Guayas estuary is located at the delta of the Guayas River Basin, covers an area of 78 km^2^ of the downstream Guayas River, and includes the Puná island beach. The study area was chosen based on the red crab distribution and the important artisanal red crab fisheries associations in the area. The Gulf of Guayaquil contains 81% of the Ecuadorian mangrove system [39]. The main economic activities in the Guayas River Basin are agriculture, fisheries, and hydropower generation [40].

### 2.2. Sampling Methodology

**Figure 1:**
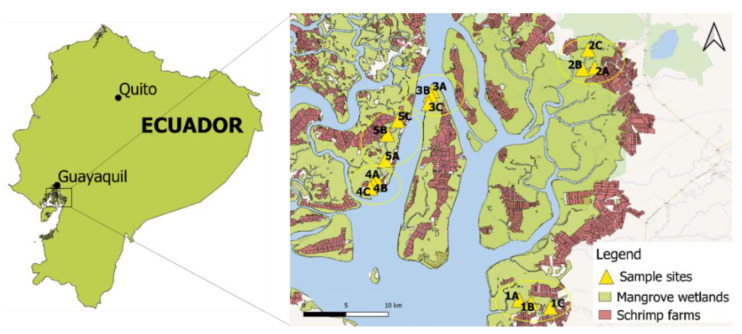
Map of the study area with indication of the sampling sites in Guayas Estuarine Basin, Ecuador.

Sampling was performed in July 2016 at five sampling stations (S1, S2, S3, S4, and S5), which were catching sites for red crab fisheries in the Guayas Estuary (Figure 1). Each station was divided into three different sampling sites (A, B, and C) to consider regional variability per station. Distance between the subsampled points ranged between 100 and 500 m. Station 1 was selected because its location is close to mining activities at Ponce Enriquez. Metal pressure was expected to be higher than station 2. Station 2 is located in the Manglar Churute Ecological Reserve, which is a protected area. However, an agricultural influence of rice fields might be present. Stations 3, 4, and 5 were selected as they are located next to the main river channel, with frequent maritime traffic, coming from the city of Guayaquil. A trend of dilution in the metal pressures was expected for these three locations because of the influx of marine waters.

A total of twelve male crabs were caught at each station by local fishermen while sediment and yellow mangrove leaf samples were taken from the exposed intertidal zone through handpicking. Only male crabs were selected, since local legislation prohibits the capture of female crabs. Sediment samples were taken from the upper sediment layer (0–10 cm) at the same locations of crab harvest in the intertidal mangrove area. Fallen leaf samples were picked from the surface of the mangrove sediments (litter). At each site, two self-closing polyethylene plastic bags (17.7 cm × 19.5 cm, SCJohnson, Guayaquil, Ecuador) were filled for 75%, one with sediment and the other with mangrove leaves. All plastic bags were hermetically closed and stored at −20 °C until transfer. Surface water samples were taken from the boat near the shore. Three samples of 0.25 L per station were stored in a pre-cleaned plastic bottle that was submerged in the top water layer (0–30 cm). All water samples were acidified with concentrated nitric acid to achieve a pH lower than 2 and frozen at −20 °C. Prior to freezing at −20 °C, a knife was inserted through the rostrum of the crabs. Then, the crabs were washed with distilled water, weighed, and the carapax width was measured (Table S1), while taking note of any abnormalities (i.e., missing limbs). The samples of crabs, sediment, leaves, and water were transferred to Belgium in a frozen state for further processing and analysis.

### 2.3. Sample Preparation

From each crab, three different tissues were extracted for analysis. The first tissue of interest was the hepatopancreas due to being the primary vessel for detoxification, storage, and metabolization of metals in crustaceans [41,42]. Unfortunately, four samples from different stations appeared to be unusable due to unclear coding. Secondly, the carapax was selected for analysis to investigate metal accumulation in this matrix as a possible detoxification mechanism of the red mangrove crab [43]. Finally, the white meat was extracted from the crab limbs for the human health exposure assessment.

The crabs were washed, and excess mud was removed carefully. First, the crab body was opened manually, and the dark yellow hepatopancreas tissue was extracted with tweezers and put it in a glass beaker. Then, all legs were pulled out and carefully crushed to extract the white meat with tweezers and collect it in a glass beaker. Each meat and hepatopancreas sample was homogenized with an Ultra-Turrax (T 25 ultra Turrax, IKA-Werke GmbH, Staufen, Germany) for 10 min, carefully avoiding cross-contamination by cleaning all material with acetone after each homogenization. The remaining carapace from the legs was cleaned thoroughly, placed in a polyethylene plastic bags (17.7 cm × 19.5 cm, SCJohnson, Ecuador), and pulverized with a hammer. Finally, all samples were weighed according to the required weight for the analysis and stored in falcon tubes at −20 °C. The sediment samples were stirred, dried at 40 °C until constant weight, and subsequently ground. Leaf samples were ground and dried at 40 °C until constant weight. The required weight of the sediment and leaf samples was weighed into falcon tubes. Before analysis, the crab tissue samples were dried at 105 °C until constant weight and, additionally, the dry weight of the hepatopancreas and white meat samples was determined.

### 2.4. Metal Analysis

For crab tissue and leaves, approximately 0.5 g was transferred into a digestion vessel; then, 10 mL of concentrated HNO_3_ was added. To prevent memory effects for Hg and improve signal stability with inductively coupled plasma (ICP) measurements, 50 µL of 1000 mg/L Au (as AuCl_3_) was added [44]. Afterwards, microwave destruction in closed Teflon liners was performed, using a MARS-6 microwave digester (CEM Corporation, Matthews, NC, USA). After destruction, the digestate was diluted to 50 mL. For sediment samples, aqua regia extraction was performed by adding 2.5 mL 68% HNO_3_ and 7.5 mL 38% HCl to 1 g of sample [45]. The solution was left at 21 °C for 12 h. Afterwards, the suspension was heated at 150 °C for 2 h. Subsequently, the digestate was diluted to 100 mL. Two mL of 86% HNO_3_ and 1 mL of 30% H_2_O_2_ was added to the 50 mL of water samples. The mixture was boiled under reflux on a hot plate. Afterwards, HNO_3_ and H_2_O_2_ were added until all suspended matter disappeared. After destruction, the solutions were diluted to 50 mL.

In the extracts, the metals Cu, Ni, Cr, As, Pb, Cd, and Hg were determined using inductively coupled plasma mass spectrometry (ICP-MS Elan DRC-e, PerkinElmer, Waltham, MA, USA). Zinc was analyzed using inductively coupled plasma optical emission spectrometry (ICP-OES Vista-PRO, Agilent Technologies, Santa Clara, CA, USA). The data are reported in µg/kg dry weight (dw) and were converted to µg/kg fresh weight (fw) prior to the consumers’ risk assessment.

### 2.5. Environmental Evaluation Based on National and International Indices

To obtain an indication of the health and bio-safety of the mangrove ecosystem related to the observed concentrations of metals, the biota-sediment accumulation factor (BSAF), geo-accumulation Index (Igeo), and hazard quotients (HQ) were calculated.

The BSAF estimates the relationship between a bioaccumulated contaminant in an organism and the contaminant concentration in the sediment. This factor is a desirable method to assess the accumulation of the pollutant in the organism [46]. Equation (1) was used to estimate the BSAF.

(1) $$\mathrm{BSAF}=\frac{Cx}{Cs}$$

where *Cx* is the total concentration of a certain metal in the crab and *Cs* is the concentration of the metal in the sediment, both being expressed in the same units.

Equation (2) was used to calculate the Igeo factor, which gives an indication of the grade of sediment contamination [47].

(2) $$\mathrm{Igeo}\;=\;\log2\left(\frac{Cs}{1.5*Bn}\right)$$

where *Cs* is the concentration of the metal in the sediment and *Bn* is the geochemical background concentration of the same metal.

The HQ presents an indication of the hazard the contaminants might present to the aquatic environment compared to an environmental quality standard (*EQS*). As no national legislation regarding metals in sediment was available, we set the *EQS* for sediment as the Probable Effect Level threshold (PEL) reported by Canadian Council of Ministers of the Environment (CCME) [48,49]. This threshold value represents the probable effect level and indicates the metal concentration in sediment above which harmful effects are likely to occur to aquatic life. The *EQS* for water was set as the National Recommended Water Quality Criteria, the Criterion Continuous Concentration (CCC) in saltwater [50]. Both quality guidelines were chosen, since they are frequently used in other studies. Equations (3) and (4) were used to calculate the HQ in sediment and water, respectively.

(3) $$\;\mathrm{HQs}=\frac{Cs}{EQS}$$

(4) $$\mathrm{HQw}=\frac{Cw}{EQS}$$

where *Cs* is the concentration of the metal in sediments and *Cw* is the concentration of the metal in water. Furthermore, the metal concentrations in water were compared to the national water threshold values for the protection of aquatic life [51].

### 2.6. Consumer Health Risk Assessment

#### 2.6.1. Exposure Assessment

In the current research, a probabilistic chronic exposure assessment was performed. Two datasets are required to perform an exposure assessment: the distribution of contamination of the hazard at the moment of consumption and the distribution of the consumption information for the targeted population [52]. The concentration data of the metals in the crab tissue (white meat and hepatopancreas) (µg/kg fresh weight) was combined with discrete distributions based on crab consumption data published by the Division of Science, Research and Technology, New Jersey Department of Environmental Protection (NJDEP) [53,54] (Tables S2 and S3). Calculations were carried out using the software of @Risk for Microsoft Excel version 7.6.0 [55]. Probabilistic models obtain a more realistic metal intake estimation, since any variability or uncertainty in variables is reflected in the model output compared to a deterministic approach [56]. The exposure level was assessed based on the total metal concentrations measured in the crabs, i.e., no adjustments were made for intra-individual correlations, bio-accessibility of the metals [57], or for a potential degradation of the compounds during cooking of the crabs. Therefore, this approach may result in a potential overestimation of exposure. Additionally, the metal concentrations below the Limit of Quantification (LOQ) were set equal to LOQ, to provide insight into the worst-case scenario (upper bound scenario). Furthermore, two cases were analyzed, the first one assuming the consumer only eats the white meat of the red mangrove crab, while in the second case, we assumed the consumer eats the hepatopancreas and white meat of the red mangrove crab. Distributions were fitted to the metal concentrations of both cases, and a discrete distribution was fitted to the consumption data published by NJDEP. Best-fit distributions were selected based on Chi-square statistics. Furthermore, the probability/probability plots (P/P) and the quantile/quantile plots (Q/Q), gave insight in the conformity of the cumulative distributions to the theoretical cumulative distributions. As children are in general more sensitive to contaminants than adults, the exposure assessments for each case were calculated on the one hand applying the children’s body weight (bw = 30 kg) and on the other hand adopting the adult body weight (bw = 65 kg) [57]. First-order Monte Carlo simulations were performed with 1000 iterations to simulate the exposure per metal. The estimated exposures (mean, standard deviation, and percentiles) were determined for each metal separately.

#### 2.6.2. Risk Characterization

The results of the exposure assessment for Zn, Cu, Ni, Cr, Cd, and Hg were evaluated with reference to the tolerable daily intake values (TDIs) as set by EFSA and FAO/WHO (Table 1). Exposures above the TDIs were considered of public health concern. Regarding the metals As and Pb, the previously set provisional maximum tolerable daily intake (PMTDI) values were withdrawn, since these values were not considered as health-protective according to the Joint FAO/WHO Expert Committee on Food Additives (JEFCA) [58,59]. The margin of exposure (MOE) is defined as the ratio of the benchmark dose lower confidence limit (BMDL) for the critical effect to the predicted exposure dose or concentration of the metal [60,61]. Regarding the MOE for Pb, EFSA reported that for effects on systolic blood pressure (SBP) or kidney in adults and neurodevelopmental toxicity, MOE values of ≥ 10 “should be sufficient to ensure that there was no appreciable risk” [58]. The current study analyzed the total As concentration in the red mangrove crab; however, health threshold values are based on inorganic As (In-As). Since In-As is a carcinogenic and genotoxic contaminant, exposure to inorganic As should be as low as reasonably practicable. We performed a literature search to determine a ratio of Inorganic As/Total As. Organoarsenical compounds constituted the vast majority of total arsenic found in sea foods and are mostly non-toxic; up to 90% of As in fish muscle is present in the non-toxic arsenobetain form [59,62,63]. Based on literature studying arsenic in crabs, we found a range of 0.5 to 5% for the In-As content in total As [64,65,66]. Based on previous studies, we assumed in this research that 1% of the total As consists of In-As. This percentage was multiplied with the As concentrations in the crab tissues. Consequently, a probabilistic risk assessment was performed on the theoretical In-As concentrations in the red mangrove crab. The MOE for inorganic As should be >10,000 to be considered as low concern from a public health point of view and to be reasonably considered as a low priority for risk management actions [59,60,67]. Finally, a threshold value for the intake of the red mangrove crab to protect human health (adults and children) was established based on the theoretical residue concentrations of inorganic As concentrations (Equations (5) and (6)).

(5) $$\mathrm{EXPlim}\;=\frac{BMDL_{01}}{MOE}$$

(6) $$\mathrm{Clim}\;=\frac{EXPlim}{Res}*a*b*c$$

with *EXPlim* being the upper limit for As exposure when *MOE* = 10,000 and *BMDL_01_* = 0.3 μg/kg·bw/day, *Clim* being the maximum crab intake without potential chronic health risk for consumers, *Res* being the probabilistic In-As residue concentration (μg/kg), *a* being a conversion factor of 0.026 crab/g crab meat, *b being* an adult body weight of 65 kg or a children body weight of 30 kg, and *c* being the number of days in a month. Based on the exposure results, assumptions on a potential health risk for crab consumers were made.

### 2.7. Data Analysis

All statistical analyses were conducted with R Version 1.2.5033 [73]. The Shapiro normality test was conducted on the metal concentration datasets. Non-parametric tests were used since the metal data were not normally distributed. The Kruskal–Wallis test (non-parametric) followed by the post-hoc Dunn test with Bonferroni correction was performed to detect variability/significant differences between the metal concentrations in the different compartments for the different stations. All statistical tests were evaluated at a significance level of 0.05. We applied a worst-case scenario for all analyses/presentations of the data by setting all concentrations below the LOQ equal to the LOQ. The sampling sites were graphically illustrated using QGIS version 2.18.4.

## 3. Results

### 3.1. Metals in Environmental and Crab Matrix

#### 3.1.1. Environmental Components

**Figure 2:**
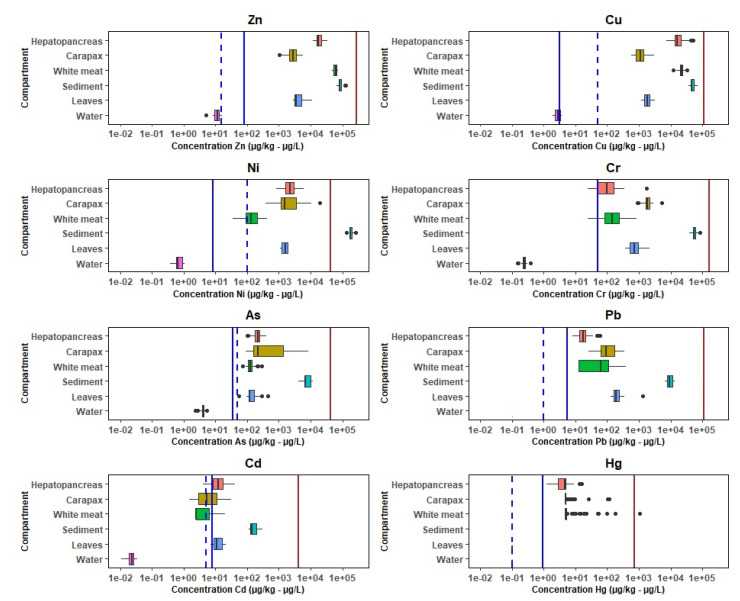
Boxplots with distribution of each metal in the compartments (µg/L for water compartment, and for others µg/g dry weight ). Indication of Probable Effect Level threshold for sediment quality (PEL) in brown, Criterion Continuous Concentration (CCC) for water quality in blue, and the national legislation threshold values for metals in water in dashed blue.

All metals analyzed were present in each environmental matrix, of which each concentration appeared to be metal- and matrix-specific (Figure 2). The observed metal concentrations in water were higher compared to water of undisturbed wetlands in Arab Emirates and mangrove water in Singapore [74,75]. Only Cd values in the Guayas mangrove waters were lower than in the Singapore mangroves. Furthermore, Defew et al. [76] reported average values of several metal(loid)s in mangrove sediments and leaves around the world (Contaminated mangroves: Panama, Costa Rica, Colombia, Australia; and Clean mangroves: Brazil, China) [76]. When comparing the Zn and Cu concentrations in the Guayas sediment with these values, it is observed that the concentrations in this study are intermediate. In contrast, Ni and Cr concentrations appeared to be significantly higher, while Pb and Cd concentrations were significantly lower than concentrations reported by Defew et al. [76]. They also reported significantly higher metal concentrations for Zn and Cu, with one order of magnitude, in the leaves of mangroves in Punta Mala Bay (Panama). Furthermore, Defew et al. found similar Pb concentration in leaves of a clean mangrove compared to concentrations in the Guayas leaves.

#### 3.1.2. Crab Matrix

Zn had in all tissue the highest concentration as one could expect, as this element is considered essential (Figure 2) [77]. Pb, Cd, and Hg had in all tissue the lowest concentrations. For Cu, Ni, As, and Cr, the order was variable depending on the tissue. Cd is known to accumulate more in the brown meat of crabs [78], which was also the case for the red mangrove crab under study. Cadmium concentrations in the hepatopancreas were on average twice as high as in the white meat; however, all concentrations in both tissues were below the maximum level (ML) in crustaceans of 0.5 mg/kg wet weight [79]. The same MLs were set by EFSA for Pb and Hg concentrations in crustaceans, and again, the levels of Pb and Hg in the red crab white meat were below the MLs [80,81].

### 3.2. Spatial Distribution

Regarding the environmental compartments, apart from Ni in water and Cd in sediment, no significant differences were present between the five sampling stations (Table 2). This indicated a low variability of the metal concentrations in sediment, leaves, and water between the stations. However, various significant differences were present for the metal concentrations in the crab tissue between the five sampling stations. The variability between the stations is observed mainly in the metals stored in the carapax and hepatopancreas, and less in the white meat. However, there is a remarkable significant difference of Ni in white meat of crabs between station 1 and all other sampling stations. In addition, the Ni concentrations stored in the carapax of crabs differed significantly between stations.

### 3.3. Metal Pollution in the Mangrove Wetland

A useful indication of the grade of sediment contamination is given by the Igeo. Muller has defined seven classes of geo-accumulation index ranging from Class 0 (Igeo < 0, unpolluted) to Class 6 (Igeo > 5, extremely polluted) [47]. The index indicated that the metals Zn, Cu, Cd, Pb, and Cr are present in natural concentrations (Table 3). Yet, it also indicated that the mangrove sediments were low to moderately contaminated with Ni and As. All BSAF values were < 1, indicating that no bio-accumulation of the contaminants occurs in the red mangrove crabs [46]. With respect to the hazard quotients, the HQ for Ni in sediment indicates an intermediate pollution that can lead to fatal effects to sensitive organisms (HQ = 4.6). Brix et al. identified that nickel potentially causes reductions in growth and reproduction and/or alterations in energy metabolism in aquatic organisms [82]. As for the other metals, the HQ was < 1, indicating natural levels of metals at all stations with no or reversible effect on aquatic organisms. Of all metals, only the Ni concentrations in the Guayas mangrove sediment exceeded the PEL quality limit. All metal concentrations appeared to be below the CCC and national water quality guidelines (Figure 2).

### 3.4. Consumer Health Risk Assessment

There was no significant difference between the exposure values for metal concentrations in only the white crabmeat and the exposure values combining the metal concentrations in the hepatopancreas and white meat (*p* > 0.05). In addition, no significant difference was found between the exposure results for children and adults. The results indicate that the crab-consuming population (children and adults) is exposed to the metals Zn, Cu, Ni, Cd, and Hg at levels that do not exceed the toxicological reference values (Table 4). It can be observed that standard deviations are higher than the mean exposure values; this could be due to the variability found in the metal concentrations in the hepatopancreas and white meat between the five stations. The probabilistic exposure distribution was below the TDI, which indicates that zero percent of the crab-consuming population is at risk regarding Zn, Cu, Ni, Cd, and Hg. With respect to the MOE value for Pb, no appreciable risk is observed since the MOE values for adults and children were higher than 10. After performing the risk assessment for In-As, the exposure data indicated a medium concern for food safety (1000 < MOE < 10,000). The MOE demonstrated that a chronic health risk might have been developed in the consumers of the red mangrove crab. Long-term exposure to In-As in the environment can lead to increased risks of skin, lung, bladder, and/or kidney cancer in humans [83]. Based on the assumptions that were made, upper-limits (Clim) of eight crabs per month for adults and four crabs per month for children were established to avoid potential human health risks in the long term when only crab meat is consumed. In case the white meat and hepatopancreas is consumed, a limit of four crabs per month for adults and two crabs per month for children is suggested. Importantly, the drying of samples at high temperatures might result in the evaporation of Hg [84]. Therefore, in this study, a potential underestimation of Hg concentrations in the crab tissues occurred.

## 4. Discussion

### 4.1. Metal Distribution in the Mangrove Wetlands

In general, the metal presence in Ecuador and elsewhere in the world is linked to natural causes such as volcanic eruptions and volcanic rocks (taking into account the presence of the Andean mountain range) and anthropogenic sources related to domestic, agricultural, industrial, oil processing, and mining activities [11,12,85]. Pollution from sewage and agriculture, changes in land use, and two hydro-electrical power dams are the main environmental pressures on the freshwater ecosystems of the Guayas basin [10,40,86]. For example, Cd is known to occur in soils in Ecuador and to accumulate in significant amounts in certain crops such as cacao and rice [13,87]. This phenomenon was not observed in the case of the red mangrove crab and its ecosystem. The Igeo index indicated that Zn, Cu, Cd, Pb, and Cr appeared to be present in natural concentration levels, indicating that the mangrove ecosystem has not been significantly contaminated by anthropogenic sources. In addition, no national guidelines regarding metals in water were exceeded (Figure 2). 

Trace metals are known to be one of the most common contaminants bound to estuarine sediments [88]. These elements are also a natural part of the environment, albeit in very low concentrations. The general neutral or slightly alkaline environment of rivers ensures that metals are mainly associated with the sediment [89]. Compared to the PEL threshold, Ni appeared in elevated concentrations in the mangrove sediments. These results comply with previous studies in which high Ni levels were observed in estuaries due to the inherent intermediate salinity. As a consequence, aquatic biota will be most at risk from Ni in these habitats [90,91]. However, in the case of the red mangrove crab living in the Guayas sediment, the BSAF value indicates no bioaccumulation, indicating these organisms are less susceptible to adverse health effects due to Ni.

Furthermore, the Igeo index indicated a moderate contamination of As in the mangrove sediment. However, it must be observed that the Igeo index uses general background values and does not consider the high contribution of volcanic rock to the presence and concentrations of As in soils and sediment in Ecuador.

Arsenic and Ni are widely distributed in nature as a result of environmental sources (e.g., gold ores and volcanic activity) and anthropogenic pollution, which is to a great extent caused by smelting of nonferrous elements, glass manufacturing, thermal power plants using fossil fuels, agriculture, and fertilizers [92,93,94,95]. In addition, industrial mining activities are active near the estuarine system and could be a potential source of As and Ni contamination [96]. Further research is needed to investigate the sources of metals, specifically Ni and As contamination, in the Guayas estuary.

### 4.2. Spatial Distribution

The distribution of the different metals in the crab tissue between the different stations was perceived to be significantly different for certain stations, depending on the crab tissue. On the contrary, no significant differences between the stations were observed for the metal concentrations in sediment, water, and leaves, apart from Ni in water and Cd in sediment. These metal concentrations in the sediment, leaves, and water of the Guayas mangroves were more homogeneously distributed than expected, in contrast to the different pollution inputs at each station (Section 2.2). The results indicated that apart from the metal concentrations in water, sediment, and leaves, most probably other factors play a role in the bioaccumulation of the different metals in the crab tissues. For example, these factors could be the metal bioavailability, crab age and feeding habits, local biophysical factors, micro-climate, other stress factors (pesticides, organochlorine compounds, polychlorinated biphenyls), and sediment granulometry. Previous research also mentioned that a direct relationship between metal concentrations in sediment and biota was often masked by other influencing factors in their study [97]. More investigation is needed to discover and understand the metal accumulation mechanisms that operate in the crab and what are the factors that influence the pathways for metal uptake. Furthermore, as the sampling campaign was established once and completely during the dry period (July and August), no rain events were observed, and no comparison of the metal concentrations over time could be performed. The latter could be particularly important for the concentrations in the water, whereas we consider the concentrations in the sediment, leaves, and crabs to be more stable. Previous studies reported the influence of the season on the metal concentrations in sediment and crabs [98,99,100,101]. However, Van Ael et al. [97] reported that sediment and biota metal concentrations in their research were not influenced by season. Therefore, it is suggested to sample crabs, sediment, and leaves twice a year and water at regular intervals during the year to include temporal variation. Moreover, tidal effects are not taken into account in this research but important to consider, as a previous study reported that relatively higher concentrations of metals were found at high tide compared to low tide due to, among other things, redox condition, presence of hydroxides, and oxyhydroxides [102]. So, it is suggested to sample at low and high tide to include variations due to the tidal cycle.

### 4.3. Health Risk Assessment

The risk assessment exposure results indicated a potential risk for consumers of the red mangrove crab due to the presence of As. Inorganic arsenic has been classified as a carcinogenic agent by the International Agency for Research on Cancer [59,103], as long-term exposure to inorganic arsenic can induce various cancers [65,83]. Consumption of contaminated food and drinking water is the major route of exposure to As in humans [77]. For instance, previous studies reported that the consumption of rice in Ecuador is the main route of As ingestion [104]. Considering the potential uptake of inorganic As through the consumption of red mangrove crabs, rice, and drinking water, awareness has to be raised on the health of the local population. Based on the assumptions made, we advise to apply the derived limit of eight crabs per month for adults and four crabs per month for children for consumers of crab white meat. Importantly, in certain regions, the consumer is used to eating the white meat and the hepatopancreas of the crab; in this case, only half of the suggested limit is advised. It is important to consider that the combined intake of contaminated rice, water, and crabs should lead to a lower number of crabs to be consumed due to the additive exposure. It should be taken into account that the cooking process of crabs might have a positive or negative effect on the concentrations and bio-availability of the metals in the crab [105,106,107]. Further research is advised on the effect of the cooking process on the metal concentrations, especially As, in the red mangrove crab. Regarding Hg in seafood, researchers mostly focus on one Hg compound in particular, which is methylmercury (MeHg) [108]. The bioaccumulation of this toxic substance leads to concentrations in fish over a million times greater than surrounding waters, which results in detrimental impacts to aquatic life and wildlife and consequently on humans [109]. Therefore, research will be performed to determine the MeHg concentrations in the red mangrove crab to be able to evaluate the consumers’ risk considering this toxic compound.

### 4.4. Sustainable Development in the Estuarine Basin

The assessment of metal distributions in agricultural soils and aquatic ecosystems and the identification and management of associated risks and impacts is crucial to achieve the United Nations Sustainable Development Goals and to prevent adverse environmental and health impacts [9,22,30,31]. Moreover, aquatic ecosystems are one of the most threatened ecosystems in the world, which results in the decline of aquatic biodiversity and fisheries ([110]. As the Guayas River Basin is turning more and more industrial and agricultural activities are incrementing, aquatic and human life need to be protected. By analyzing pollutants and determining the distribution, adequate action, mostly prevention, can be taken regarding these threatening pollutants. Investigation on the source of these contaminants in the estuary and action to reduce this contamination or eliminate the input of these contaminants is needed. This will lead to the protection of plants, animals, and biodiversity below water and on the land. Furthermore, the red mangrove crab is a species of major commercial importance. It is not only part of the national culture as a local delicacy, but the livelihood of thousands of fishermen depends on it. In the Gulf of Guayaquil, 13% of the families depend directly on the harvest of the red mangrove crab [29]. As fishing is a major economic activity in the Guayas Basin that sustains food security and provides livelihoods for their people, a link with the following goals can be identified: good health (SDG 3), no poverty (SDG 1), zero hunger (SDG 2), improving water quality (SDG 6), decent work and economic growth for fishermen (SDG 8), promoting of local culture and products (SDG 12), increasing scientific knowledge to protect marine biodiversity (SDG14), and promoting sustainable use of wetlands and the protection of threatened species (SDG15) [111]. Finally, as any intervention cannot be accomplished without the development of solid partnerships and collaborations, with this initiative, the relations between Ghent University and the local partner ESPOL (Escuela Superior Politécnica del Litoral) are strengthened in order to accomplish an agenda of sustainable development in Ecuador (SDG 17). The presented results raise awareness about the contamination of precious ecosystems and the consequence for environmental and human health.

## 5. Conclusions

The dispersion of conceivably toxic metals in aquatic ecosystems and related food is a public health concern of global urgency. As metals do not degrade, the detected elements Zn, Cu, Ni, Cr, As, Pb, Cd, and Hg remain in the mangrove life cycle for a long time and end up in the red mangrove crabs. Elevated Ni concentrations in the mangrove sediments could lead to potential adverse health effects for sensible aquatic organisms. The risk assessment exposure results indicated a potential risk for consumers of the red mangrove crab due to the presence of As. It is advised to the consumers of crab meat to apply a consumption limit of eight crabs per month for adults and four crabs per month for children to protect their health in the long term. However, these recommended limits were set without considering additive exposure of As contaminated rice and/or water. As shown in this research, the assessment of metal distributions in aquatic ecosystems and management of associated risks are paramount regarding food safety and associated socio-economic aspects of fisheries. Further research is required regarding the sources of the metals, the metal uptake and accumulation mechanisms of the crab, along with the presence of inorganic as and methylmercury in the red mangrove crab, as this study is seen as a first exploration in this area.

## Figures and Tables

**Table 1:** Toxicological values for metals used for risk characterization, with PTWI being the provisional tolerable weekly intake and PTMI the provisional tolerable monthly intake.

Metal	Toxicological Value from Reference			Toxicological Value Tested Against	
	Type	Value	Reference	Type	Value
Zn	PMTDI	0.3–1 mg/kg·bw/day	[68]	TDI	300 µg/kg·bw/day
Cu	PMTDI	0.5 mg/kg·bw/day	[68]	TDI	500 µg/kg·bw/day
Ni	TDI	2.8 μg/kg·bw/day	[69]	TDI	13 µg/kg·bw/day
Cr	TDI	0.3 mg/kg·bw /day	[70]	TDI	300 µg/kg·bw/day
In-As	BMDL01	0.3 μg/kg·bw/day	[59]	MOE	10,000
Pb	BMDL01	12 µg/kg·bw/day	[58]	MOE	10
Cd	PTMI	25 μg/kg·bw/month	[71]	TDI	0.81 µg/kg·bw/day
Hg	PTWI	4 µg/kg·bw/week	[72]	TDI	0.57 µg/kg·bw/day

**Table 2:** Significant differences found between the metal concentrations in the biotic and abiotic compartments at the five stations.

	Zn	Cu	Ni	Cr	As	Pb	Cd	Hg
Hepatopancreas	S2 > S5 S4 > S5	S2 > S1			S2 > S1	S2 > S3 S2 > S4 S5 > S3	S3 > S4 S5 > S4	S3 > S5
Carapax	S1 > S5 S2 > S5 S3 > S5	S1 > S4 S2 > S4 S2 > S5	S3 > S1 S3 > S2 S3 > S4 S3 > S5	S3 > S1 S3 > S2 S5 > S2	S1 > S2 S1 > S4 S1 > S5 S3 > S2 S3 > S4 S3 > S5	S3 > S2 S3 > S4 S3 > S5		S1 > S3 S1 > S5
White meat		S1 > S3	S1 > S2 S1 > S3 S1 > S4 S1 > S5					
Sediment							S2 > S4	
Leaves								
Water			S1 > S3					

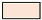
 = Significant difference between certain stations (*p* < 0.025).

**Table 3:** Results of the Igeo, BSAF, and HQ for the different metals.

Index	Matrix	Zn	Cu	Ni	As	Cd	Pb	Cr
HQs	Sediment	0.3	0.5	4.6	0.2	0.04	0.1	0.4
HQw	Water	0.0001	0.0009	0.00009	0.0001	0.000003	-	0.000005
Igeo	Sediment	−0.3	−0.7	0.8	1.5	−0.9	−1.0	−1.4
BSAF	Crab/sediment	0.9	0.1	0.2	0.2	0.2	0.02	0.04

**Table 4:** Probabilistic exposures of metals in hepatopancreas and white meat of red mangrove crab for adults (mean, standard deviation (SD), and percentiles (Px)), % TDI calculated as (estimated mean exposure/TDI) × 100; mean, SD, and percentile expressed as μg/kg·bw/day. TDI: tolerable daily intake; MOE: margin of exposure.

Metal	Mean	SD	P50	P75	P95	Population Exceeding TDI (%)	% TDI	MOE at Mean Exposure
Zn	6.97	8.19	3.96	9.60	27.1	0	2.32	-
Cu	3.26	4.03	1.74	4.25	12.2	0	0.65	-
Ni	0.15	0.20	0.07	0.19	0.56	0	1.15	-
Cr	0.022	0.036	0.010	0.027	0.100	0	0.01	-
In-As	0.0005	0.001	0.0002	0.0005	0.002	-	-	1423
Pb	0.009	0.015	0.003	0.011	0.035	-	-	55.8
Cd	0.001	0.002	0.001	0.001	0.004	0	0.15	-
Hg	0.002	0.005	0.0004	0.001	0.007	0	0.30	-

## Data Availability

Data are contained within the article or supplementary material.

## Supplementary Materials

The following are available online at https://www.mdpi.com/article/10.3390/foods10081880/s1, Figure S1: Distribution of metals in each compartment, Figure S2: Boxplots with distribution of the metals in the compartments per station, Figure S3: Boxplots with distribution of metals in each compartment, Figure S4: Boxplots with distribution of metals in water, Figure S5: Violin plots with distribution of each metal in the different compartments (except for water) per station (1, 2, 3, 4 and 5 indicating each station), Figure S6: Violin plots with distribution of each metal in water per station (1, 2, 3, 4 and 5 indicating each station), Figure S7: Radar plot showing the average distribution of the metals in the crab and environmental compartments, Figure S8: Radar plots per station showing the distribution of the metals in the crab compartments, Figure S9: Radar plots per station showing the distribution of the metals in the environmental, Figure S10: Radar plots per metal showing distribution in compartments, Table S1: Crab codes, weight and carapax length per crab, Table S2: Consumption data for health risk analysis and weight crab meat and hepatopancreas per crab (g) analyzed in the current research, Table S3: Probabilistic Residue formulas, Table S4: Metal concentrations in crab compartments and environmental compartments, Table S5: Limit of detection (LOD) and limit of quantification (LOQ) levels for the different compartments, Table S6: Recovery data for sediment reference materials, Table S7: Recovery data for crab reference material, Table S8: In-situ measurements at sample sites. DO: Dissolved oxygen, ND: Not detected, BDM: Below detection limit., Equations.
