# Supplementary material for: From Mangrove to Fork: Metal Presence in the Guayas Estuary (Ecuador) and Commercial Mangrove Crabs
Source: Foods. 2021 Aug 14;10(8):1880. doi: 10.3390/foods10081880 (PMC8393220; doi:10.3390/foods10081880)
Supplement: Supplementary file 1 [file foods-10-01880-s001.zip › foods-1294202-supplementary.pdf]

Supplementary Materials

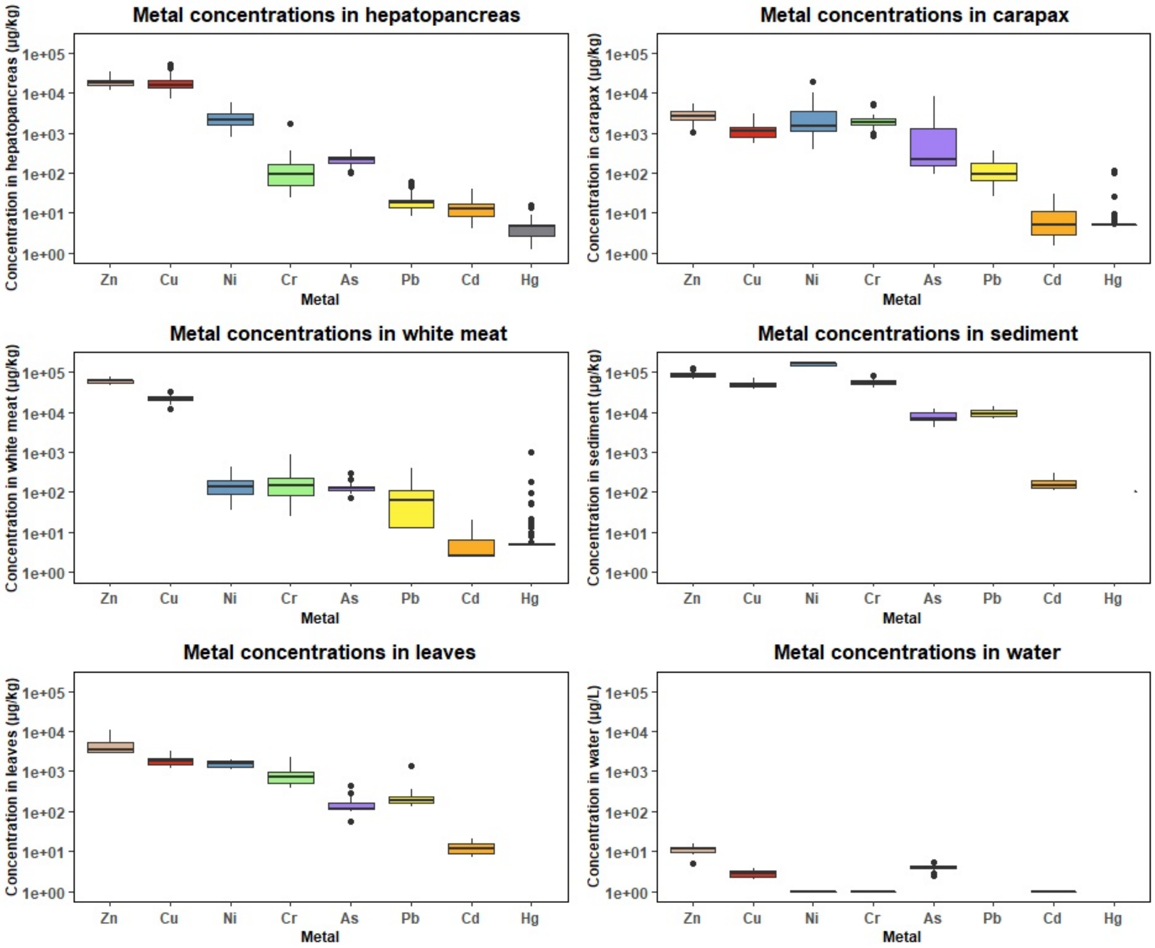

Figure S1: Distribution of metals in each compartment (µg/kg dw - µg/L). Note: Hg was not analyzed in the environmental matrices (sediment, leaves, water) and concentrations in water for Ni, Cr and Cd were <1 and Pb <LOD.

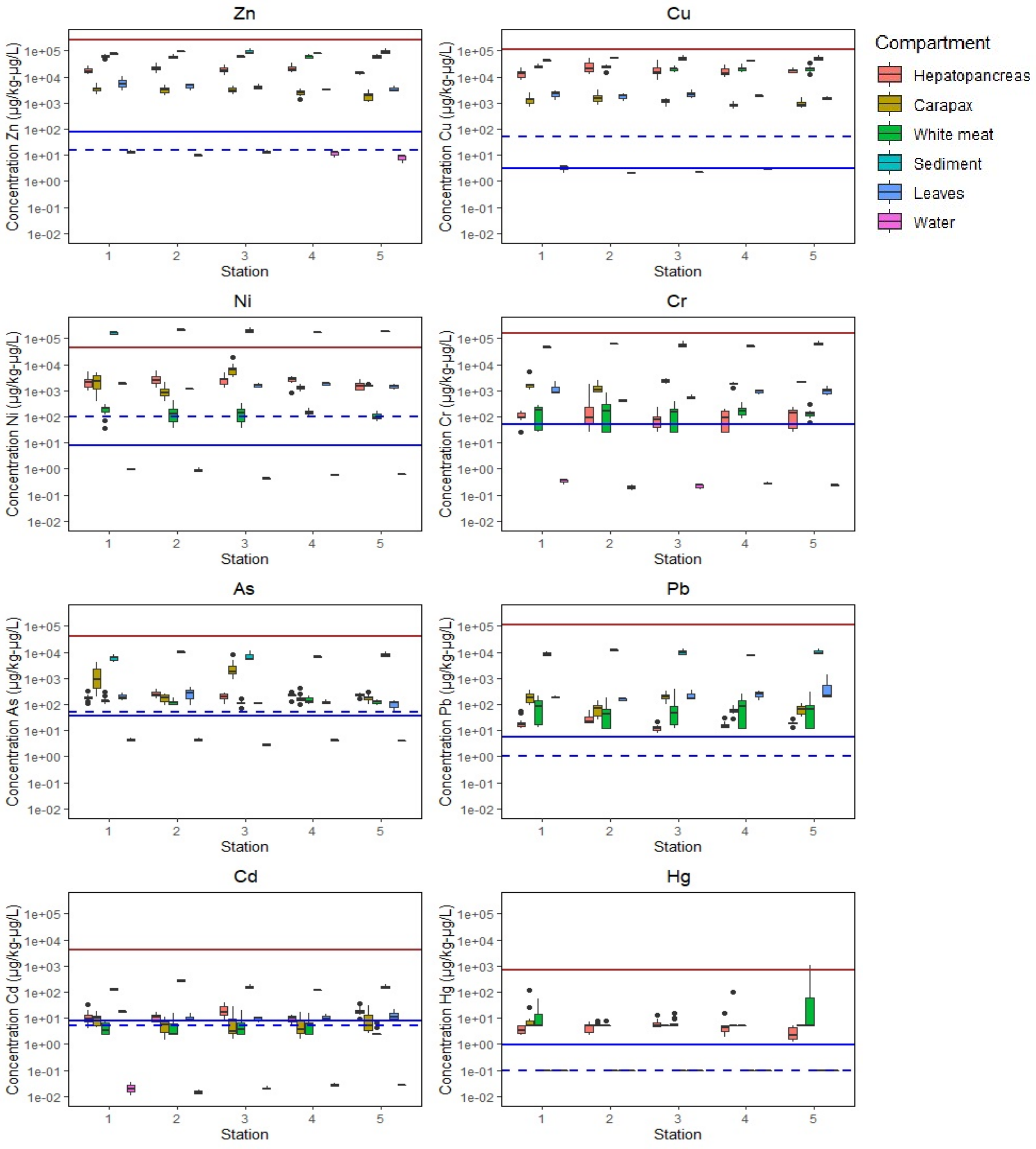

Figure S2: Boxplots with distribution of the metals in the compartments per station (µg/kg dw - µg/L). Indication of PEL in brown, CCC in blue and the Ecuadorian national legislation threshold values for metals in water in dashed blue. Note: Hg was not analyzed in the environmental matrices (sediment, leaves, water) and concentrations in water for Pb were <LOD.

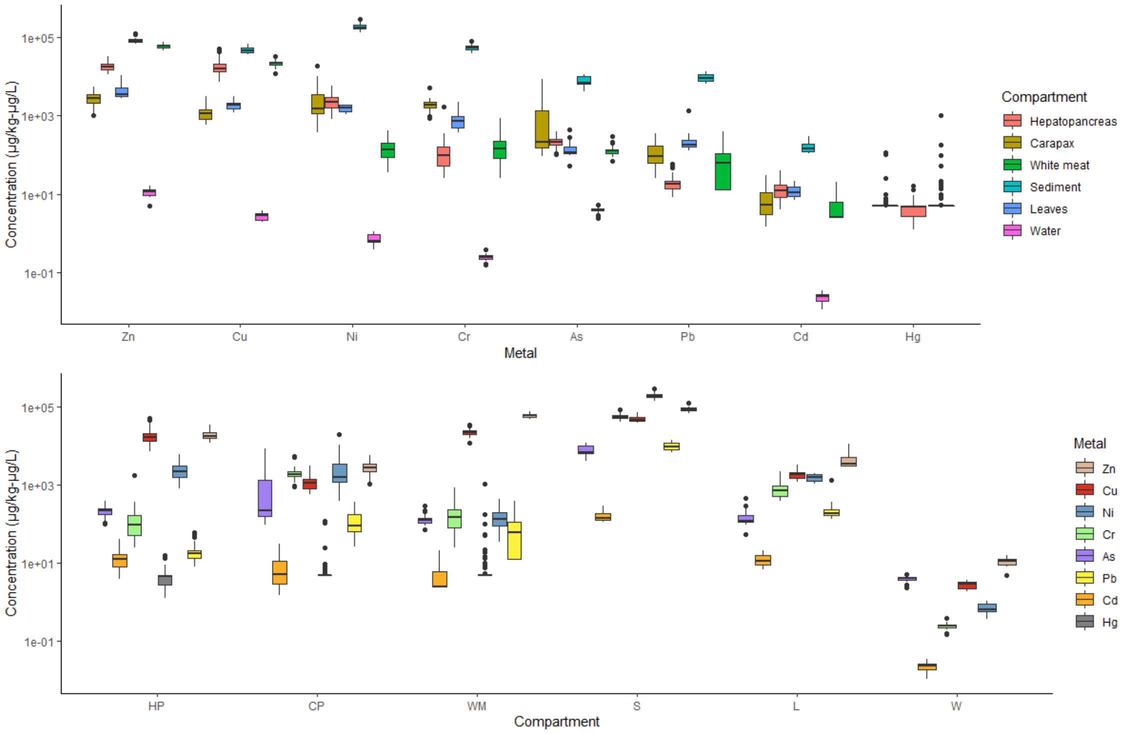

Figure S3: Boxplots with distribution of metals in each compartment (µg/kg dw - µg/L).

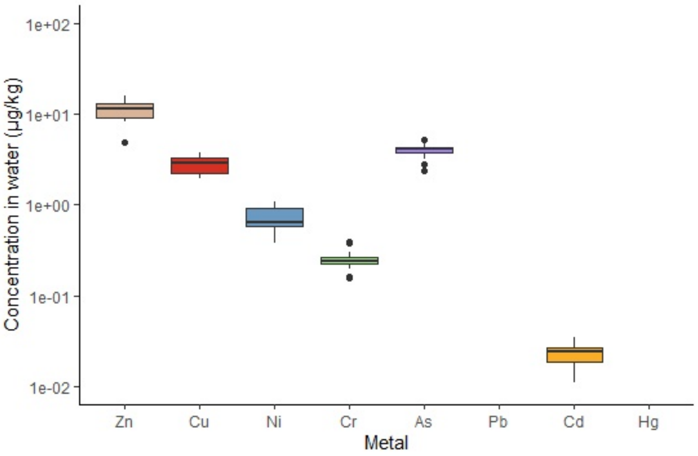

Figure S4: Boxplots with distribution of metals in water (µg/L).

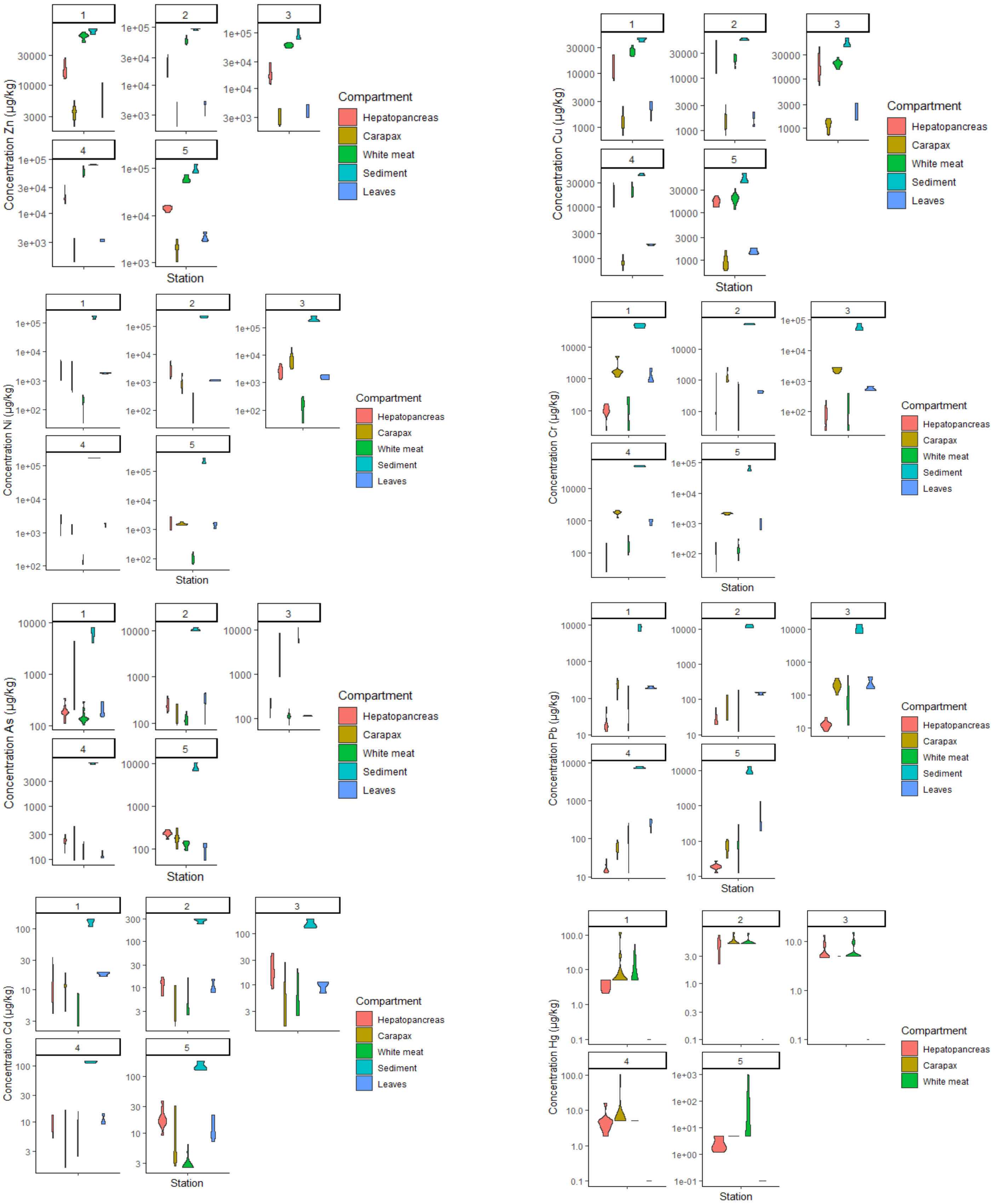

Figure S5: Violin plots with distribution of each metal in the different compartments (except for water) per station (1, 2, 3, 4 and 5 indicating each station) (µg/kg dw).

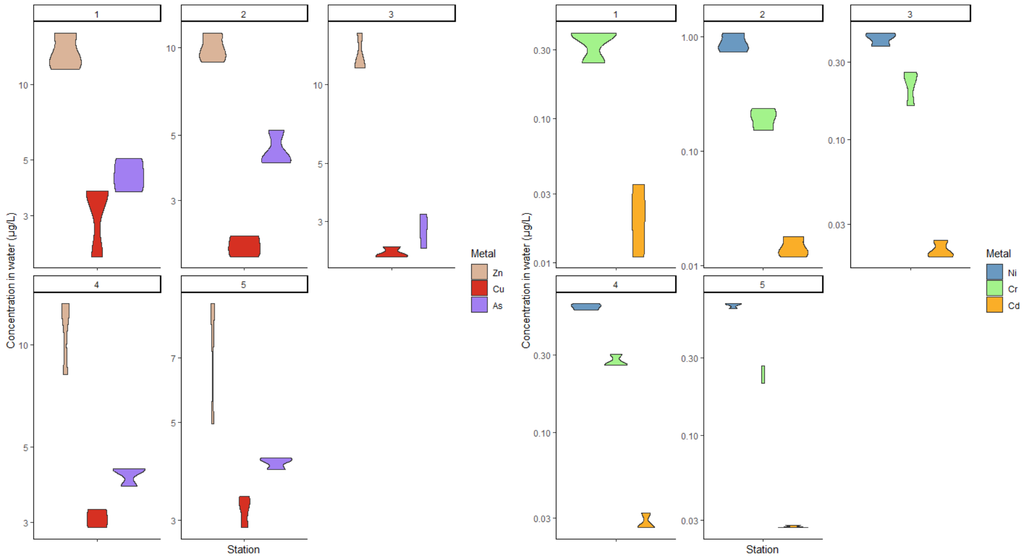

Figure S6: Violin plots with distribution of each metal in water per station (1, 2, 3, 4 and 5 indicating each station) (µg/L).

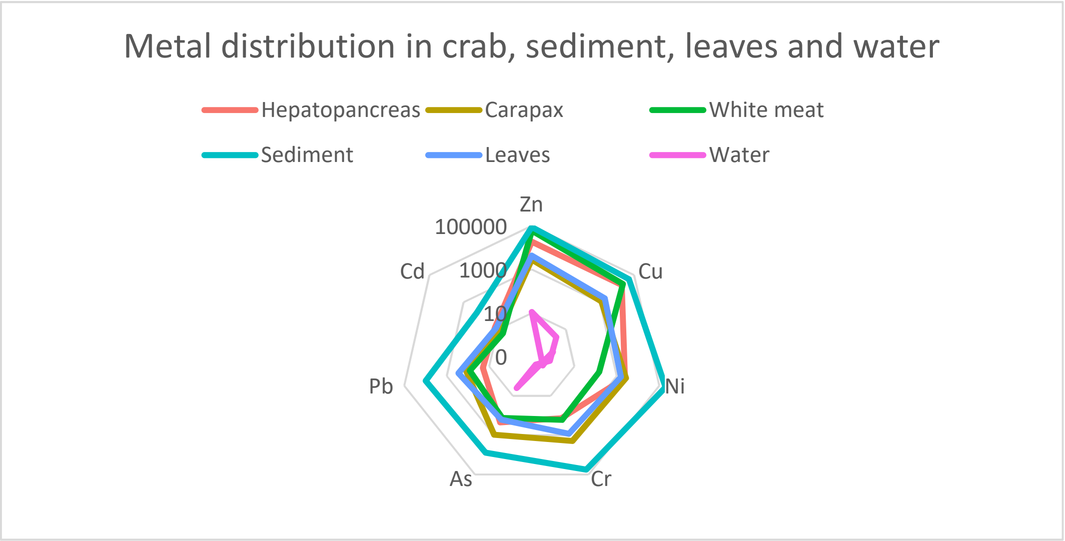

Figure S7: Radar plot showing the average distribution of the metals in the crab and environmental compartments (µg/kg dw - µg/L).

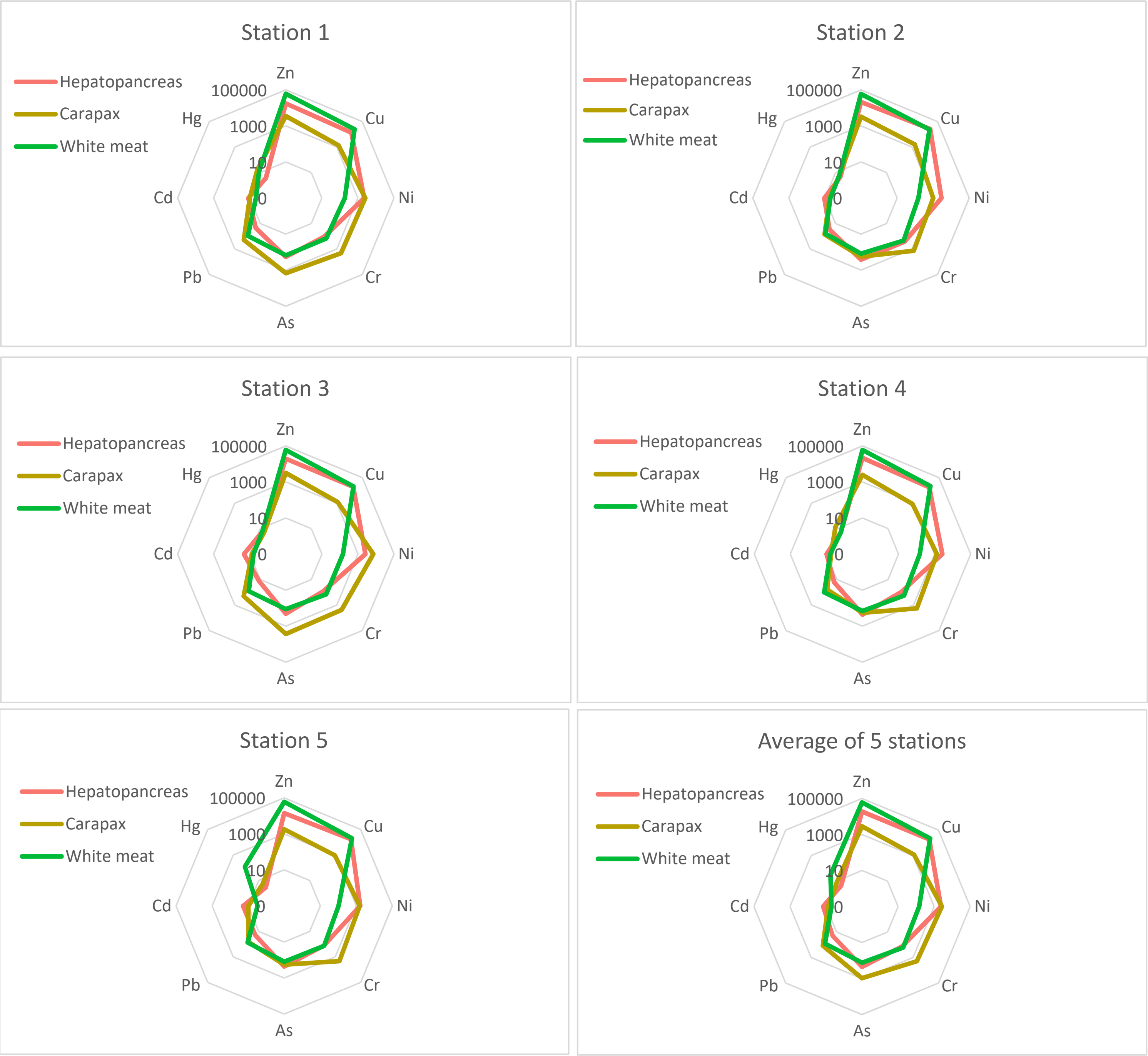

Figure S8: Radar plots per station showing the distribution of the metals in the crab compartments (µg/kg dw).

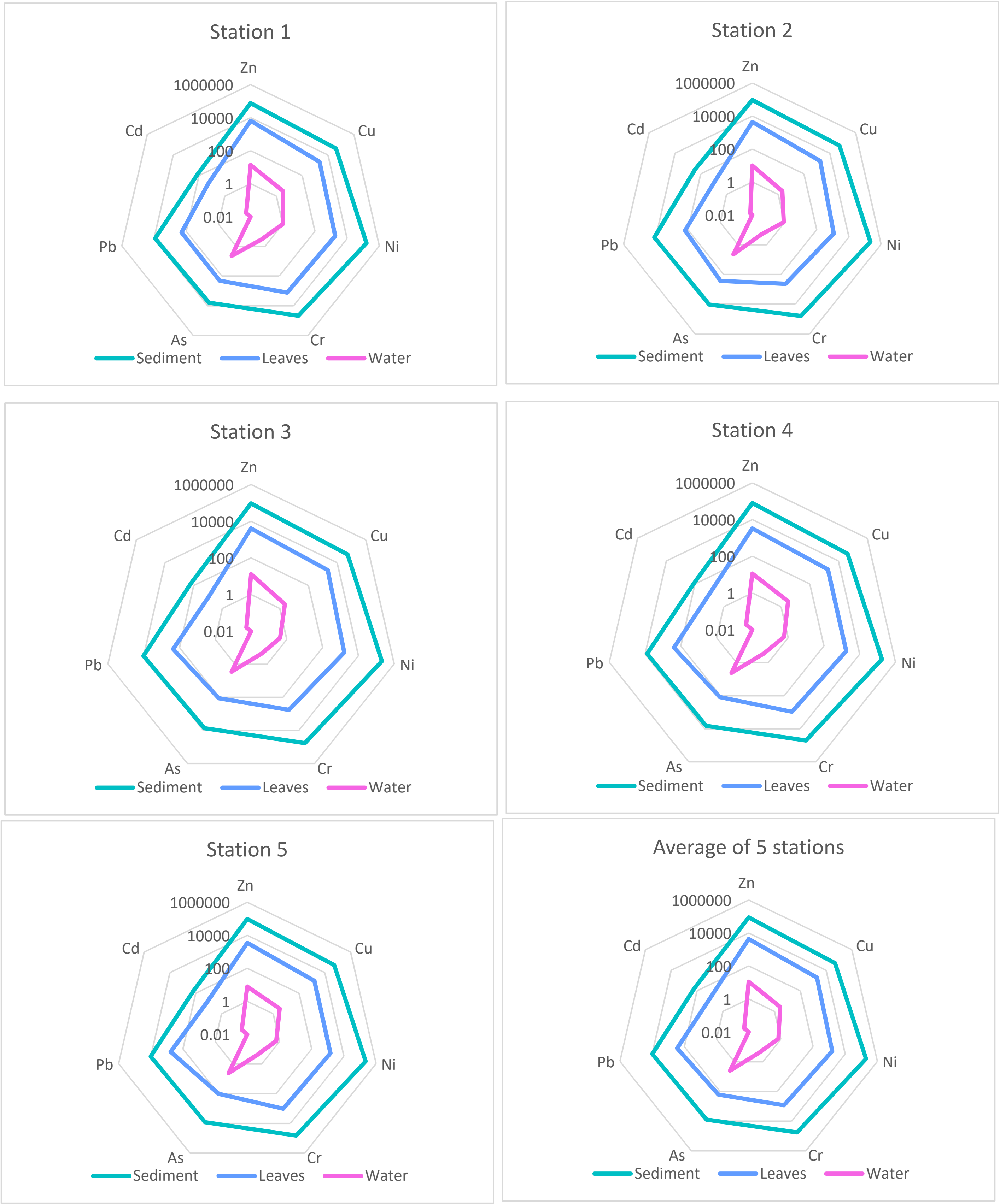

Figure S9: Radar plots per station showing the distribution of the metals in the environmental compartments (sediment and leaves µg/kg dw - water µg/L).

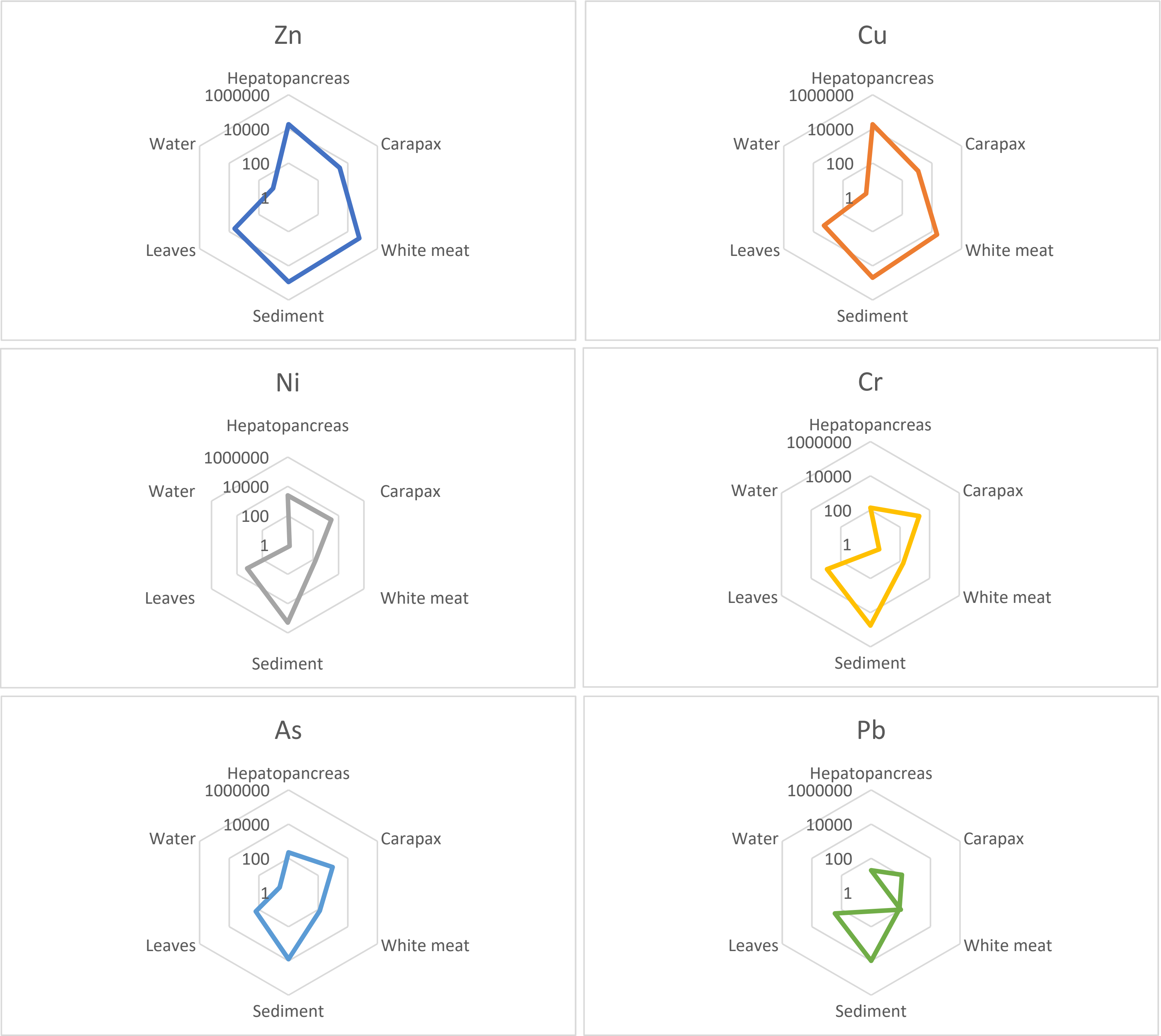

Figure S10: Radar plots per metal showing distribution in compartments (crab, sediment, and leaves µg/kg dw - water µg/L).

Table S1: Crab codes, weight and carapax length per crab.

Station	Crab Code	Weight (kg)	Carapax length (cm)	Station	Crab Code	Weight (kg)	Carapax length (cm)
S1	1/1	0.24	92	S4	4/1	0.21	82
	1/2	0.24	86		4/2	0.25	85
	1/3	0.20	84		4/3	0.21	85
	1/4	0.23	86		4/4	0.26	93
	1/5	0.22	82		4/5	0.16	77
	1/6	0.22	83		4/6	0.21	81
	1/7	0.22	89		4/7	0.22	85
	1/8	0.22	88		4/8	0.17	79
	1/9	0.21	82		4/9	0.15	79
	1/10	0.19	80		4/10	0.22	79
	1/11	0.26	89		4/11	0.19	78
	1/12	0.23	83		4/12	0.15	73
S2	2/1	0.23	92	S5	5/1	0.22	83
	2/2	0.17	79		5/2	0.27	87
	2/3	0.21	83		5/3	0.23	84
	2/4	0.20	83		5/4	0.21	80
	2/5	0.21	85		5/5	0.19	83
	2/6	0.18	85		5/6	0.20	78
	2/7	0.20	81		5/7	0.17	83
	2/8	0.17	84		5/8	0.20	88
	2/9	0.16	77		5/9	0.19	76
	2/10	0.16	82		5/10	0.19	77
	2/11	0.18	82		5/11	0.21	81
	2/12	0.17	84		5/12	0.20	84
S3	3/1	0.14	78				
	3/2	0.20	80				
	3/3	0.19	81				
	3/4	0.18	80				
	3/5	0.19	81				
	3/6	0.17	80				
	3/7	0.18	75				
	3/8	0.20	79				
	3/9	0.17	77				
	3/10	0.13	72				
	3/11	0.18	75				
	3/12	0.17	77				

Table S2: Consumption data for health risk analysis (Source: the Division of Science, Research and Technology, New Jersey Department of Environmental Protection (NJDEP, 2002; Pflugh et al., 2011) and weight crabmeat and hepatopancreas per crab (g) analyzed in the current research.

Consumption data

	**Meal per day**	**Percentage of consumers**
Every day	1	0.03
2 to 3 times a week	0.36	0.23
Once a week	0.14	0.20
Twice a month	0.07	0.20
Once a month	0.03	0.12
Less than once a month	0.02	0.13

	**Number crabs per meal**	**Percentage of consumers**
1-3 crab	2	0.27
4-6 crab	5	0.29
7-10 crab	9	0.08
11-15 crab	13	0.19
More 15 crab	18	0.08

**Weight crabmeat and hepatopancreas per crab (g)**	**Percentage of crabs**
26	0.02
27	0.02
28	0.07
33	0.08
34	0.05
35	0.02
36	0.08
37	0.05
38	0.10
39	0.19
40	0.08
41	0.03
42	0.08
43	0.02
44	0.03
45	0.02
47	0.03
50	0.02

Table S3: Probabilistic Residue formulas

**Metal**	**Probabilistic residue concentration(ug/kg)**
**Cr**	=RiskExtvalue(23,291;16,782;RiskName("Cr"))
**Ni**	=RiskLoglogistic(-11,239;211,29;4,328;RiskName("Ni"))
**Cu**	=RiskWeibull(1,7132;2002,9;RiskShift(2814,8);RiskName("Cu"))
**Cd**	=RiskLognorm(1,4731;0,77435;RiskShift(0,37926);RiskName("Cd"))
**Hg**	=RiskInvgauss(1,5216;0,24118;RiskShift(0,78834);RiskName("Hg"))
**Zn**	=RiskLoglogistic(4429,4;5277,8;7,3177;RiskName("Zn"))
**As**	=RiskLaplace(35,1632;7,1264;RiskName("As"))
**As Inorg**	=RiskLaplace(35,1632;7,1264;RiskName("As"))*RiskDiscrete(I25:I27;J25:J27;RiskName("Discrete distribution In-As"))/100
**Pb**	=RiskGamma(0,70949;15,127;RiskShift(2,6332);RiskName("Pb"))

Table S4: Metal concentrations in crab compartments and environmental compartments. LOD: Limit of detection

	HP (mg/kg dw)		CP (mg/kg dw)		WM (mg/kg dw)		S (mg/kg dw)		L (mg/kg dw)		W (µg/L)	
	Average	Range	Average	Range	Average	Range	Average	Range	Average	Range	Average	Range
Zn	19±5	12-34	3±1	1-6	60±7	48-75	88±16	67-126	4±2	3-11	5-16	0.005-0.02
Cu	19±9	7-52	1±0.5	1-3	22±4	12-33	50±10	37-70	2±1	1-3	2-4	0.002-0.004
Ni	2±1	1-6	3±3	0.4-19	0.1±0.1	0.04-0.4	196±41	137-294	2±0.3	1-2	0.4-1.1	0.0004-0.001
As	0.1±0.2	0.03-2	2±0.8	0.9-5	0.2±0.1	0.03-0.9	8±2	4-12	0.2±0.1	0.1-0.4	2.4-5.2	0.002-0.005
Cd	0.2±0.1	0.1-0.4	1±2	0.1-8	0.1±0.04	0.07-0.3	0.17±0.06	0.1-0.3	0.01±0.005	0.007-0.02	0.01-0.04	0.00001-0.00004
Pb	0.02±0.01	0.01-0.06	0.1±0.1	0.03-0.4	0.1±0.1	0.01-0.4	10±2	7-14	0.3±0.3	0.1-1.3	<LOD	<LOD
Hg	0.01±0.008	0.004-0.04	0.008±0.006	0.001-0.03	0.005±0.004	0.003-0.02	NA	NA	NA	NA	NA	NA
Cr	0.004±0.003	0.001-0.02	0.009±0.02	0.005-0.1	0.03±0.1	0.01-1	57±12	39-83	0.8±0.5	0.4-2.2	0.2-0.4	0.0002-0.0004

Table S5: Limit of detection (LOD) and limit of quantification (LOQ) levels for the different compartments.

Metal	WATER		LEAVES		SEDIMENT		CRAB	
	LOD (µg/L)	LOQ (µg/L)	LOD (µg/kg)	LOQ (µg/kg)	LOD (µg/kg)	LOQ (µg/kg)	LOD (µg/kg)	LOQ (µg/kg)
**Cr**	0.02	0.06	0.17	0.56	1.68	5.60	0.17	25.00
**Ni**	0.04	0.14	0.43	1.43	4.29	14.30	0.43	35.00
**Cu**	0.04	0.12	0.35	1.17	3.51	11.70	0.35	1.17
**As**	0.01	0.04	0.13	0.42	1.25	4.17	0.13	0.42
**Cd**	0.00	0.01	0.03	0.11	0.33	1.10	0.03	2.50
**Pb**	0.00	0.00	0.01	0.05	0.14	0.47	0.01	12.50
**Hg**	0.03	0.10	0.30	1.00	3.00	10.00	0.30	5.00
**Zn**	1.76	5.87	18.00	60.00	176.00	586.67	18.00	60.00

Table S6: Recovery data for sediment reference materials.

Element	BCR 277 (estuarine sediment)[1]		CRM 052 (Loamy clay)[2]	
	Recovery (%)	Uncertainty reference material (%)	Recovery (%)	Uncertainty reference material (%)
Cd 114	78	11	136	3
Cr-KED 52	85	7	121	1
Ni-KED 58	212	6	124	3
Zn-KED 64	77	11	0	0
Cu-KED 65	84	11	86	2
As-KED 75	89	10	102	2

[1] (Evisa, 2010a)

[2] (Evisa, 2010b)

Table S7: Recovery data for crab reference material.

Element	TORT-2 (lobster hepatopancreas)[1]	
	Recovery (%)	Uncertainty reference material (%)
Pb-totaal 208	95	37
As-KED 75	77	8
Ni-KED 58	93	8
Cu-KED 65	83	9
Cr-KED 53	105	19
Cd-KED 114	74	2
Hg 202	102	22
Zn	95	3

[1] (Evisa, 2010c)

Table S8: In-situ measurements at sample sites. DO: Dissolved oxygen, ND: Not detected, BDM: Below detection limit.

Sample ID	Conductivity (µS/cm)	pH (-)	Temperature (°C)	DO (mg/L)	DO Sat (%)	Nitrate-N (mg/L)	Nitrite-N (mg/L)	Ammonium-N (mg/L)	Orthophosphate (mg/L)
1A	36.6	7.42	25.8	5.65	67.5	ND	ND	ND	ND
1B	34.7	7.3	25.8	3.83	47.2	BDL	0.052	0.272	1.46
1C	26.1	7.39	26.3	4.66	57.4	ND	ND	ND	ND
2A	6.23	7.53	26.5	5.11	63.4	BDL	0.041	0.122	0.7
2B	13.41	7.4	25.9	4.83	59.4	ND	ND	ND	ND
2C	12.68	7.37	25.6	4.23	51.7	BDL	0.036	0.039	0.69
3A	10.96	7.48	26.5	6.68	83.3	0.9	0.029	0.051	0.57
3B	13.69	7.45	26.4	6.36	78.8	1.3	0.042	0.116	0.64
3C	15.94	7.45	26.3	6.36	87.8	0.6	0.045	0.146	0.61
4A	32.3	7.64	26.9	6.96	87.4	BDL	0.022	0.002	0.36
4B	33	7.61	26.7	6.86	85.5	BDL	0.026	0.068	0.3
4C	34.6	7.67	26.5	6.97	86.6	0.6	0.023	0.008	0.31
5A	30.8	7.63	26.6	6.7	83.3	BDL	0.025	0.055	0.34
5B	30.7	7.44	26.7	5.8	72.3	BDL	0.04	BDL	0.31
5C	30.8	7.41	26.7	5.48	68.4	0.4	0.054	0.01	0.32

Equations

A BSAF value of <1 indicates a low bioavailability of the metal and no bio-accumulation in the crab. A BSAF of 1 < BSAF < 2 indicates that the organism is a micro-concentrator, and a BSAF value > 2 indicates that the organism is a macro-concentrator.

The factor of 1.5 in Igeo is introduced to minimize the effect of possible variations in the background or control values which may be attributed to lithogenic variations in the sediment (Barbieri, 2016; Okedeyi et al., 2014). According to the abundance data in the Earth's Crust of Krauskopf and Bird (1995) the Bn values were Zn:70, Ni:75, Cr:100, Pb:13, Cu:55, Cd:0.2, and As:1.8 mg/kg (Hasan et al., 2013; Krauskopf & Bird, 1995). Muller (1979) has defined seven classes of Geo-accumulation Index ranging from Class 0 (Igeo<0, unpolluted) to Class 6 (Igeo>5, extremely polluted) (Muller, 1979).

It is reported by Samara et al. that a value of HQ < 1 refers to unpolluted sites with no or reversible effect on aquatic organisms, whereas a value of HQ > 1, indicates a potential ecological hazard, 1 < HQ < 2 indicates low pollutant load with no acute danger for organisms; 2 < HQ < 10 indicates intermediate pollution that can lead to fatal effects to sensitive organisms and finally HQ > 10 signifies high pollution with effects on the reduction of benthic organism diversity (Nabelkova & Kominkova, 2012; Samara et al., 2020; Sample et al., 1996).
